# Knowledge, Beliefs, and Practices on Epilepsy among High School Students of Central Nepal

**DOI:** 10.1155/2017/6705807

**Published:** 2017-02-12

**Authors:** Lekhjung Thapa, Tirtha Raj Bhandari, Shakti Shrestha, Ramesh Sharma Poudel

**Affiliations:** ^1^Department of Neurology, National Institute of Neurological and Allied Sciences, Kathmandu, Nepal; ^2^Department of General Medicine, Primary Health Centre, Jutpani, Chitwan, Nepal; ^3^Department of Pharmacy, Shree Medical and Technical College, Bharatpur, Nepal; ^4^Department of Hospital Pharmacy, Chitwan Medical College Teaching Hospital, Chitwan, Nepal

## Abstract

*Introduction*. Epilepsy continues to increase worldwide but, unfortunately, many high school students have inadequate knowledge of and negative beliefs towards the disease. We aimed to assess the knowledge, beliefs, and practices of epilepsy among high school students of Central Nepal.* Materials and Methods*. A cross-sectional study was performed involving 1360 high school students from 33 private schools across Bharatpur, from June 2013 to July 2013, to assess their knowledge, beliefs, and practices (KBP) on epilepsy using a standardized questionnaire. The differences in mean KBP scores between different sexes, religions, and those personally knowing versus not knowing someone with epilepsy were assessed using independent *t*-tests; a Pearson correlation was calculated to assess the relationship between KBP scores and age.* Results*. Of 1360 participants, 79 (5.8%) students had never heard or read about epilepsy and were consequently excluded from statistical analysis. Only 261 out of 1360 (19.2%) had personally known someone with epilepsy. The mean KBP scores were 5.0/8, 7.4/12, and 1.7/3, respectively. Statistically significant differences were only observed in the knowledge component of the KBP score; female scored higher than males (*p* < 0.001) and, interestingly, students who had personally known a person with epilepsy actually knew less than those who had not known one (*p* = 0.018). We also found a significant negative correlation between knowledge and age (*p* = 0.003).* Conclusions*. The overall knowledge, beliefs, and practices appear to be inadequate, emphasizing the need for further educational intervention.

## 1. Introduction

Epilepsy is a chronic brain disorder characterized by recurrent derangement of the nervous system due to sudden excessive disorderly discharge of the aggregate group of neurons from cerebrum. The excessive discharges result in disturbances of sensation, convulsive movement, or psychic function with or without loss of consciousness [1]. Epilepsy affects all age groups but is more common in children. The reported prevalence of epilepsy in developing countries is 5 to 10 per 1000 people. Global prevalence is 2.8 to 19.5 per 1000 people [1]. Currently epilepsy affects 50 million people worldwide, of which 80% live in developing countries [2–4].

Social stigma and discrimination often cause more suffering for people with epilepsy than the seizures themselves [5]. People living with epilepsy are discriminated against in all facets of life, from education to employment and marriage [1]. Although the etiology of stigma and discrimination is complex, lack of the knowledge regarding epilepsy is purported to be an important determinant of negative attitudes. Children with epilepsy, especially those who have seizures at school, suffer from discrimination and report feeling different from their peers. They also have fear of suffering a seizure at school [5].

Historically, epilepsy was believed to be a sacred disease resulting from invasion of the body by a god; it was thought that only god could deprive a healthy man of his senses, throw him to the ground, convulse him, and then rapidly restore him to consciousness [3]. Many people in developing countries believe that epilepsy is contagious and spreads via urine, saliva, flatus and faeces excreted during a convulsion. This belief has played a major role in people living with epilepsy being ostracized, stigmatized, and misunderstood [3].

Understanding knowledge, beliefs, and practices with respect to epilepsy in the community is the first important step in forming strategies to dispel the myths and misconceptions regarding the disease [5]. Educating children about the reality of epilepsy is critical in alleviating the stigma faced by children with epilepsy at school. Moreover, children are the future workforce and have the potential to become role models in society. The progressive emergence of positive public beliefs towards people with epilepsy has been demonstrated in recently conducted KBP surveys in both developed and developing countries [5].

KBP surveys comparing developing versus developed countries have demonstrated a significant knowledge and beliefs gap among high school students towards their peers with epilepsy.

Generally speaking, students from developed countries have superior knowledge and hold more positive attitudes towards children with epilepsy, while negative beliefs and bias remain a major concern for children with epilepsy in the developing world.

Bharatpur is situated in the mid southern part of Nepal and is more developed when compared with the majority of other Nepalese districts. This is evidenced by a higher literacy rate and life expectancy rate as well as better health and transportation facilities. We designed this study to quantify and characterize KBP with respect to epilepsy among high school (Grade 10) children in Bharatpur.

## 2. Materials and Methods

### 2.1. Study Design

This cross-sectional study assessed the KBP on epilepsy among 1360 high school students (Grade 10) and was conducted in all 33 private schools of Bharatpur, from June 2013 to July 2013. Written consent was obtained from each school and participant. The participating students were given verbal guidance on how to fill out the questionnaire. Students studying in government schools were not included in the survey due to more difficult access when compared to private schools.

### 2.2. Questionnaire

The questionnaire included 25 prevalidated KBP questions (Tables 2 and 3): 2 questions on familiarity with epilepsy, 8 questions on knowledge, 12 questions on beliefs, and 3 questions on practices. This was modified from a study performed in South India [5]. The questionnaire also included demographic questions. The response to KBP questions was binary—either “yes” or “no.” Correct (positive) responses scored one point whereas incorrect (negative) responses scored zero points. The highest possible scores for knowledge (K-score), beliefs (B-score), and practices (P-score) were eight, twelve, and three, respectively. Health professionals oriented to the questionnaire collected the data. The permission for data collection was sought from the college principal. All students were instructed to complete the questionnaire and questions were clarified as needed.

### 2.3. Statistical Analysis

All statistical analysis was performed using IBM-SPSS 20.0 (IBM Corporation, Armonk, NY, USA). Students who had never heard/read about epilepsy were excluded from statistical analysis of the KBP scores. Descriptive statistics were ascertained for all variables. The mean differences in KBP scores were compared between categories of sexes (male and female), between categories of religions (Hindu and others), and between students who personally knew and had not known someone with epilepsy using independent *t*-tests. A Pearson correlation coefficient was calculated to assess the relationship between KBP score and age. Statistical significance was taken when *p* < 0.05.

## 3. Results

### 3.1. Sociodemographic Characteristics

A total of 1360 students (mean age 15.2 ± 0.8 years) completed the questionnaire. There were 730 (55.9%) boys and 600 (44.1%) girls. Very few students (22, 1.6%) had a family history of epilepsy and most of the students (1241, 91.3%) did not have a neighbour with a history of epilepsy (Table 1).

### 3.2. Familiarity with Epilepsy

The majority of students (1281, 94.2%) had heard/read about epilepsy but very few of them (261, 19.2%) had personally known someone with epilepsy (Table 2).

### 3.3. Knowledge about Epilepsy

**Figure 1:**
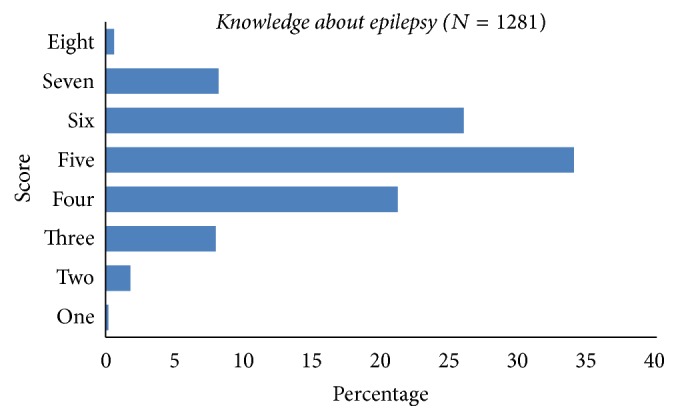
Knowledge scores of high school students about epilepsy.

The majority of high school students responded correctly to the eight knowledge questions about epilepsy (Q(1) to Q(4) and Q(13) to Q(16)); refer to Table 3. Most participants knew that epilepsy is neither a contagious disease (881, 68.8%), nor a hereditary disease (829, 64.7%), nor a mental disease (730, 57.0%). However, greater than half (695, 54.3%) responded that epilepsy is not a disease of the brain. Similarly, most of the participants were aware that allopathic treatment is beneficial for epilepsy (1098, 85.7%) but in contrast about two-third of them (884, 69.0%) were unaware that Ayurvedic treatment is not beneficial for epilepsy (Table 3). The mean ± standard deviation (SD) knowledge score was 5.01 ± 1.17 and the percent of each score is shown in Figure 1.

### 3.4. Beliefs Concerning Epilepsy

**Figure 2:**
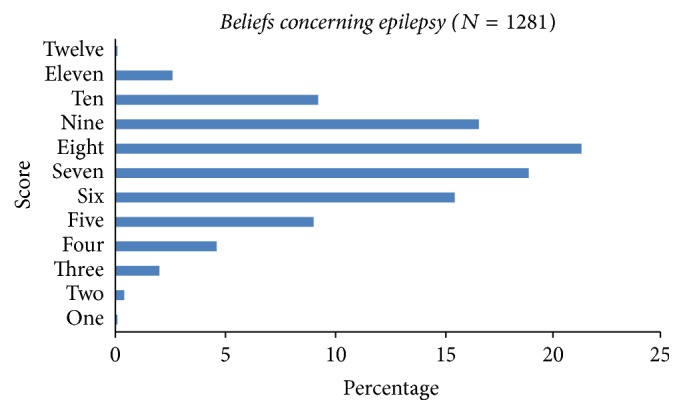
Beliefs scores of high school students concerning epilepsy.

The twelve questions of beliefs about epilepsy (Q(5) to Q(12) and Q(17) and Q(20)) are shown in Table 3. The majority of the response was positive, although most students believed epilepsy to be a hindrance to a happy life (844, 65%) and would affect a person's education (751, 58.6%). There were 731 (57.1%) students who felt that the society should not discriminate against people with epilepsy and 930 (72.6%) would not object to sitting in the classroom adjacent to a child with epilepsy or playing with an affected child (Table 3). The mean ± SD beliefs score was 7.35 ± 1.84 and the percent of each score is shown in Figure 2.

### 3.5. Seizure First Aid Practices

**Figure 3:**
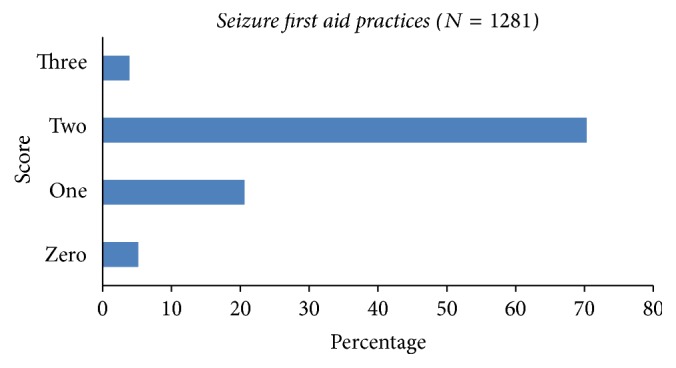
Seizure first aid practices scores of high school students.

The seizure first aid practices were assessed through Q(21a), Q(21b), and Q(21c) (Table 3). The mean ± SD practices score was 1.73 ± 0.62. The majority of students scored 2 points (900, 70.3%), followed by one point in 264 (20.6) (Figure 3). There were 236 (18.4%) participants who would not take a person fitting to hospital right away; 25.5% would sprinkle water over the face of such person and 20.1% would make such person hold a bunch of keys (Table 3).

### 3.6. Influence of Person Who Had Known Someone with Epilepsy on KBP Response

The person who had not known someone with epilepsy had slight better mean knowledge score (5.05 versus 4.85), beliefs score (7.39 versus 7.18), and practices score (1.74 versus 1.67). However, statistical significance was only found between mean knowledge score at *p* = 0.018 (Table 4).

### 3.7. Influence of Sex on KBP Response

Female students had slightly better overall KBP scores than their male counterparts but a statistically significant difference was only observed between their knowledge score (5.23 versus 4.83, *p* < 0.001) (Table 5).

### 3.8. Influence of Religion on KBP Response

Majority of students were Hindu and all other religions (Buddhist, Christian, and Muslim) were recategorised as “others” for the purposes of statistical analysis. There was no statistically significant difference on KBP scores among Hindu and other religions though knowledge score (5.02 versus 4.95) and beliefs score (7.37 versus 7.21) were slightly higher among Hindus whereas practices scores were slightly higher among other religions (1.72 versus 1.76) (Table 6).

### 3.9. Influence of Age on KBP Response

The correlation between age and knowledge was statistically significant at *p* = 0.003 though the coefficient was low and negative (−0.082) (Table 7), suggesting that knowledge score decreases with increasing age.

## 4. Discussion

Our study is one of the largest inquiries on KBP with respect to epilepsy among high school students from South Asia. Though nearly all the students were familiar with epilepsy, misconceptions about negative beliefs towards epilepsy were highly prevalent.

### 4.1. Familiarity with Epilepsy

The higher familiarity of the high school students with epilepsy observed in our study (94%) was found to be similar to that of studies done in South India (98%) [5], Canada (100%) [6], and Malaysia (87%) [7]. By comparison, a very low percentage (52%) of adolescents surveyed in United States were familiar with epilepsy [8]. The high school students in the US study were more familiar with health conditions that are found primarily in adults, such as arthritis and breast cancer, than they were with epilepsy. However, they were more familiar with conditions that are less prevalent than epilepsy, such as HIV/AIDS and Parkinson's disease [8].

### 4.2. Knowledge about Epilepsy

In our study, less than half (43%) of the students thought that epilepsy was a form of mental disease. A similar result was observed in India (59%) [5] and Nigeria (51.9%) [9]. In contrast, a higher proportion of students thought this in Tanzania (90%) [10] and Italy (64%) [11] versus a lower proportion in Cameroon (14.3%) [12], United States (19%) [8], and Canada (9%) [6]. Nearly half (45.7%) of the students considered epilepsy as a brain disease in our study. Our student had better knowledge as compared to Cameroon (18.5%, 25.7%) [12, 13] and Egypt (8.5%) [14] in identifying epilepsy as a brain disease. More than two-thirds (64.7%) of our participants thought that epilepsy was a hereditary disease. This proportion was higher than that of India (34%) [5], Cameroon (12.4%, 25.3%) [12, 13], Italy (20%) [11], and Nigeria (22.5%) [9] and lower than that of Malaysia (67%) [6]. The proportion of our participants who believed that epilepsy is a contagious disease (68.8%) was higher than that of India (14%) [5], Cameroon (57.96%, 49.89%) [12, 13], Italy (17%) [11], Nigeria (40.6%) [9], and Yemen (2.1%) [15]. More than four-fifths (85.7%) of our students preferred allopathic medicines to treat epilepsy, but 31% believed in Ayurvedic treatment. Conversely, in the Indian study, which evaluated students in the state of Kerala, allopathic treatment was preferred by just over half, but nearly three-fifths believed in Ayurvedic treatment [5]. Therefore, our Nepalese students had better knowledge on the proper method/mode of treatment of epilepsy when compared to India. Furthermore, such beliefs in traditional and alternative treatments are one of the key factors for the large treatment gap reported in developing countries, as patients seek indigenous treatments despite the availability of modern therapies [16]. About 69% of students in our study thought that patients with epilepsy required a long-term treatment as compared to 77.2% by Joshi et al. [17] and 35% by Pandian et al. in Kerala [5]. Seventy-nine percent of our students believed that medication needs to be taken regularly to prevent harm, which was higher when compared to Kerala (60.9%). Overall, our study revealed that high school students still have deficient accurate knowledge regarding the awareness of epilepsy. This could be improved through school-based health education programme [18].

### 4.3. Beliefs concerning Epilepsy

Although our study showed high school students were more familiar with epilepsy in comparison to developed countries, their beliefs were far more pessimistic. Around one-third of respondents believed that patients with epilepsy could not be happily married and employed. Conversely, the Kerala study showed that higher number of students had positive beliefs towards married life (60%) than towards employment (30%) [5], while a survey among Canadian university students showed that 84% of them expressed favourable beliefs towards both [6]. However, our study showed similar results with the Kerala study [5] on the positive beliefs towards the impact of epilepsy on education of a person. Our participants objected to sitting near or playing with a child suffering from epilepsy comparatively more (27%) than those of South India (13%) [5] and Canada (5%) [6]. Few of the students of Bharatpur (7%) believed epilepsy is an ancestor's sin compared to the students of Kerala (14%) [5]. Furthermore, 22% of Keralan students [5] versus only 8% of Bharatpur students believe that exorcism helps to drive away epilepsy from the body. These beliefs might also contribute towards the treatment gap seen in developing countries. Around 20% of the students of Bharatpur and around 50% of the students of Kerala [5] believed that epilepsy is incurable. This may contribute towards noncompliance of epilepsy medication. Finally, approximately half to two-thirds of the students of Kerala [5] and Bharatpur believed that people suffering from epilepsy had difficulty living in society.

### 4.4. Seizure First Aid Practices

The overall seizure first aid practices among high school students in our study seems satisfactory but most of the students (about 80%) would take a person with ongoing seizure to hospital right away. A study done on first aid epilepsy management in North India found nearly 74% of the students would call a doctor as first aid measure for seizure in a person with epilepsy [19]. However, nearly a quarter of them would make such person hold a bunch of keys and sprinkle water over the face, which is not a practice that would be expected in case of seizures. This signifies that there is still a need to improve incorrect practices among high school students. In contrast to this practices status, a similar study in South India reported these practices were believed less [5]. However, this study has considered taking a person with an epileptic attack to hospital as a positive response and most students agreed that they would do so. We considered that taking someone with ongoing seizure to hospital right away is not a positive first aid practice.

### 4.5. Influence of Person Who Had Known Someone with Epilepsy and Sex on KBP Response

Our study showed that the KBP score in those who do not personally know someone with epilepsy is better than those who do, but this influence is only statistically significant with regard to knowledge. In contrast to our findings, a community-based study in Ethiopia revealed that knowing someone with epilepsy is positively associated with the level of knowledge and practices related to epilepsy [20]. We also found that the KBP score was influenced by sex and females had slightly higher KBP scores than males. However, the difference was only statistically significant (*p* = 0.018) with respect to knowledge. A survey of Canadian college students suggested that female students were slightly more tolerant towards epilepsy than male students [6].

### 4.6. Influence of Religion and Age on KBP Scores of Epilepsy

Our study showed no significant difference in the mean KBP scores between religions although Hindus had slightly better knowledge and beliefs scores, whereas scores were slightly better among other religions. However, sociocultural differences have been known to significantly affect attitude towards a person with epilepsy [21–23].

Similarly, age was not correlated with beliefs and practices but significantly correlated with knowledge. The age range of our study population was 13 to 18 years and a negative correlation coefficient suggests that elder students had poorer knowledge than their younger colleagues although they all had a similar level of education.

Although our study explores many issues regarding KBP on epilepsy among high school Nepalese students, few limitations are noteworthy. As our study is limited to the students of a particular city (Bharatpur), it may not be entirely representative of our country. We evaluated children from private schools as we had easy access to these schools. We believe that the results of our study may have been different if children from government schools were also included because of the difference in their educational and socioeconomic background. Given the binary nature of our questions, one has a 50% chance of answering “correctly” independent of knowledge base. We included basic questions related to epilepsy in our survey that can influence better epilepsy practices in a community, but it is well known that the concepts about epilepsy are changing. By including more questions in the survey we could have explored KBP on recent concepts about epilepsy.

## 5. Conclusions

The high school students of Bharatpur were familiar with epilepsy but the overall knowledge, beliefs, and practices seem to be inadequate. Although our study results might not be generalizable to all students of Nepal, which would require further clarification, the preliminary results suggest that educational intervention programmes in the community are needed.

## Figures and Tables

**Table 1:** Sociodemographic characteristics of high school students (*N* = 1360).

Characteristics (*N* = 1360)	Categories	*n* (%)
Age^†^	13 to 18	15.2 ± 0.8

Gender	Male	760 (55.9)
	Female	600 (44.1)

Religion	Hindu	1187 (87.3)
	Buddhist	142 (10.4)
	Christian	23 (1.7)
	Muslim	7 (0.5)
	Secular	1 (0.1)

Family history of epilepsy	Yes	22 (1.6)
	No	1338 (98.4)

Neighbour with history of epilepsy	Yes	119 (8.8)
	No	1241 (91.3)

Note: ^†^mean ± SD instead of *n* (%). SD: standard deviation.

**Table 2:** Familiarity of high school students with epilepsy (*N* = 1360).

Questions	Yes, *n* (%)	No, *n* (%)
Have you ever heard/read about a disease called epilepsy?	1281 (94.2)	79 (5.8)
Do you personally know someone with epilepsy?	261 (19.2)	1099 (80.8)

**Table 3:** Positive responses of High school students of Grade 10 to KBP questions (*N* = 1281).

QN	Questions	Yes *n* (%)	Positive response
(1)	Is epilepsy a mental disease?	730 (57.0)	No
(2)	Is epilepsy a disease of the brain?	586 (45.7)	Yes
(3)	Is epilepsy a hereditary disease?	829 (64.7)	No
(4)	Is epilepsy a contagious disease?	881 (68.8)	No
(5)	Do you think epilepsy is caused by ancestor's sin?	1193 (93.1)	No
(6)	Do you think epilepsy is a hindrance to a happy life?	437 (34.1)	No
(7)	Is it possible for people with epilepsy to lead a married life?	923 (72.1)	Yes
(8)	Can people with epilepsy lead a normal sexual life?	805 (62.8)	Yes
(9)	Do you think that epilepsy affects the education of a person?	530 (41.4)	No
(10)	Do you think that epilepsy patients can be employed?	877 (68.5)	Yes
(11)	Do you think society should discriminate against persons with epilepsy?	731 (57.1)	No
(12)	Would you object to sitting in the classroom adjacent to a child with epilepsy or to playing with a child with epilepsy	930 (72.6)	No
(13)	Do you think allopathic treatment is beneficial for epilepsy?	1098 (85.7)	Yes
(14)	Do you think Ayurvedic treatment is beneficial for epilepsy?	397 (31.0)	No
(15)	Do you think epilepsy needs long-term treatment?	880 (68.7)	Yes
(16)	Do you think missing the drugs once in a while is harmful?	1016 (79.3)	Yes
(17)	Do you think most of the drugs used in epilepsy treatment cause side effects?	780 (60.9)	No
(18)	Do you think epilepsy can be cured?	1012 (79.0)	Yes
(19)	Do you think visiting religious places helps in curing epilepsy?	1096 (85.6)	No
(20)	Do you think exorcism helps to drive away epilepsy spirits from the body?	1181 (92.2)	No
(21)	What would you do if you happen to see a person getting an epileptic attack?		
	(a) Take him/her to hospital right away	236 (18.4)	No
	(b) Make him/her hold a bunch of keys	1024 (79.9)	No
	(c) Sprinkle water over his/her face	945 (74.5)	No

**Table 4:** Comparison of KBP scores in students who had known and had not known someone with epilepsy personally (*N* = 1281).

Characteristics	Do you personally know someone with epilepsy?		*p* value^t^
	*Yes*	*No*	
*Knowledge*	4.85 ± 1.16	5.05 ± 1.17	0.018^*∗*^
*Beliefs*	7.18 ± 1.20	7.39 ± 1.80	0.112
*Practices*	1.67 ± 0.63	1.74 ± 0.61	0.071

^*∗*^Significant at *p* < 0.05; ^t^Student's *t*-test.

**Table 5:** Comparison of KBP scores in male and female (*N* = 1281).

Characteristics	Sex		*p* value^t^
	*Male*	*Female*	
*Knowledge*	4.83 ± 1.19	5.23 ± 1.10	<0.001^*∗∗*^
*Beliefs*	7.33 ± 1.86	7.37 ± 1.82	0.672
*Practices*	1.71 ± 0.63	1.75 ± 0.60	0.197

^*∗∗*^Significant at *p* < 0.001; ^t^Student's *t*-test.

**Table 6:** Comparison of KBP scores according to religion.

Characteristics	Religion		*p* value^t^
	*Hindu*	*Others*	
*Knowledge*	5.02 ± 1.17	4.95 ± 1.18	0.452
*Beliefs*	7.37 ± 1.85	7.21 ± 1.81	0.307
*Practices*	1.72 ± 0.62	1.76 ± 0.59	0.431

^t^Student's *t*-test.

**Table 7:** Relationship between age and KBP scores.

Characteristics	Age	
	Correlation coefficient	*p* value^p^
*Knowledge*	−0.082	0.003^*∗*^
*Beliefs*	0.008	0.786
*Practices*	−0.004	0.874

^*∗*^Significant at *p* < 0.05; ^p^Pearson correlation.

## Abbreviations

KBP: Knowledge, beliefs, and practices
SD: Standard deviation
HIV: Human immunodeficiency virus
AIDS: Acquired immune deficiency syndrome.
